# Bourgeois queens and high stakes games in the ant *Aphaenogaster senilis*

**DOI:** 10.1186/1742-9994-6-24

**Published:** 2009-10-19

**Authors:** Adam L Cronin, Thibaud Monnin

**Affiliations:** 1 Laboratoire Écologie & Évolution CNRS UMR 7625, Université Pierre et Marie Curie, 7 quai St Bernard, Bâtiment A 7ème étage, Case 237, 75 252 Paris Cedex 05, France

## Abstract

**Background:**

Many animals face some form of conflict over reproductive opportunities. Queen selection in social insect colonies represents a high-stakes conflict where competition occurs among multiple queens for a few or a single reproductive role(s). The outcome of the contest is critical to the fitness of all colony individuals as most are sterile, and thus represents a conflict at multiple levels. *Aphaenogaster senilis *is a monogynous, monandrous, fission performing ant, in which queen selection occurs during colony fission and when replacement queens are produced to overcome orphaning. First-born queens are usually behaviourally dominant over subsequent queens, and eventually inherit the colony. We investigated the importance of physical dominance in queen selection in orphaned groups by manipulating the fighting ability of first-born queens via mandibular ablation.

**Results:**

First emerged queens were heavier than second emerged queens, performed almost all aggression, were behaviourally dominant 92% of the time, and prevailed in 76% of groups after co-existing for 16 days on average. Mandibular ablation had no effect on queen behaviour or contest outcome.

**Conclusion:**

Aggression is probably ritualised and contests are decided by workers based on relative queen fertility. First-born queens thus have an inherent advantage over second-born queens as they have more time to develop ovaries. Subordinates never retaliated against aggression from dominants and this lack of retaliation can be interpreted as a form of bourgeois strategy as dominants were almost always first-born. However, the lack of alternative reproductive options makes not-fighting effectively a form of suicide. High relatedness between full-sister queens means that subordinates may be better off sacrificing themselves than risking injury to both queens by fighting.

## Background

Conflict frequently arises in animal societies over reproduction. In many social insects, this conflict is centred on the queen because she is the sole reproductive in a colony of potentially thousands of individuals: workers cannot (or do not) reproduce themselves and only gain fitness indirectly. The identity of the queen is therefore of critical importance as the fitness of all individuals in the colony depends on her. In some species workers may have opportunities to select their queen, and might be expected to do so based on fecundity, relatedness, longevity, and minimising the delay to egg laying [1]. However, therein lies a conflict of interest at multiple levels: whereas colony level selection will favour rapid emplacement of a high quality queen, individual level selection acting on workers can select for behaviour that favours closely related queens (nepotism), while queens are presumably also under selection to fight among themselves [2,3].

Queen selection occurs in three main contexts in social insect colonies: i) after worker emergence in pleometrotic (multiple queen) assemblages [4], ii) associated with colony division by dependent colony founding (fission or budding) [3,5], and iii) during emergency queen replacement [3,6,7]. While queens clearly benefit from the elimination of rivals [8,1], killing of queens may be also in the interest of workers who seek to maximise colony efficiency and restore monogyny [2,8]. Unrestrained direct conflict between queens may be unfavourable because it entails the risk of both queens being injured or killed [9], potentially leading to colony failure. This is particularly true during emergency queen replacement because the supply of brood that can be reared into new queens is limited to that laid by the previous queen, so that colonies can ill afford losing new queens. This risk can be reduced by worker intervention in queen-queen contests, whereby workers collectively influence or decide the outcome of contests [2]. Previous studies suggest that in many cases workers decide contests by culling supernumerary queens [10,3,1], though direct competition between queens is also important [11,12,1]. Queens can therefore maximise their chances of prevailing via direct conflict (fighting) or indirect conflict designed to influence workers (eg: ritualised aggression (dominance displays) or advertisement of fertility). Where workers can influence the outcome of contests, they should act to favour queens that maximise their indirect fitness, that is queens that i) are more related, ii) are likely to survive the longest, and iii) have the highest fecundity [1,13]. Selective elimination of supernumerary queens by workers has been demonstrated based on abdomen size [14], proximity to brood [15], loss of mass [16,17], fertility [18], recent social environment [17], size [15,16] and chemical signalling of reproductive condition [19-22]. The presence of nepotism remains equivocal in social insects despite considerable investment in its study [23-25,3] (and references therein). When queens are equivalent or there is no information available to workers with respect to queen quality or relatedness, they should select for rapid resumption of laying activity by favouring first eclosing queens, as occurs in army ants [26].

*Aphaenogaster senilis *is a monogynous, monandrous ant that founds colonies by dependent colony foundation (= fission [27]). Supernumerary queens are produced as part of the colony reproductive process and also during emergency queen rearing [28,5]. In orphaned colonies, workers produce several (up to 5) new queens as replacements which fight together until monogyny is restored [7]. As this species is monandrous and monogynous, workers and replacement queens are full sisters and there is thus no option for nepotism [7]. There should therefore be no conflict of interest between workers during queen selection, and workers should favour new queens based on quality and the rapidity of replacement. Queens, on the other hand, are expected to fight between themselves, as they are more related to their own brood than their sister's brood. Chéron et al [7] demonstrated that contests during emergency queen replacement are resolved in favour of first-eclosed queens, which are physically aggressive toward, and dominant over, subsequently produced queens. They proposed that second and subsequently produced queens are produced as 'insurance' against death of the first-born queen, and that workers select the first-born queens to expedite the re-queening process. However, first-born queens also have a head-start in that they are produced on average seventeen days before subsequent queens, permitting them relatively more time to develop musculature, harden the cuticle, develop ovaries, and produce pheromones, which may give them a competitive advantage in direct and indirect contests with other queens.

Order of eclosion and any advantages relating to acquired asymmetries are correlated so that it remains unclear which factor is determinant in queen selection. We investigated this by manipulating the ability of first queens to dominate subsequent queens, by ablating a mandible of the first-born queen in experimentally orphaned colonies. In *Apis mellifera *the ablation of a mandible results in queen avoidance of other queens: mutilation of all queens yields stable polygynous colonies despite this species being strictly monogynous [29]. We infer that mutilation of the first-born queen in *A. senilis *should increase the proportion of second-born queens inheriting the colony if queen choice is based on phenotypic characteristics such as fighting ability, whereas there will be no effect of the treatment if queen choice is based on order of emergence. Mutilation also allows testing whether *A. senilis *queens show evidence of self-assessment, as has been argued for *Apis mellifera *[29]. Specifically, we expect that, i) if fighting is the primary means of queen selection, then ablated queens will have a lower success rate in treatment groups; ii) if fighting is ritualised (a dominance display) and queens are instead selected by workers, ablation may have no effect on queen success; iii) These outcomes may be influenced by the behaviour of ablated queens, which we expect to be less aggressive and more evasive if self-assessment is occurring.

## Methods

Twenty-nine colonies of *A. senilis *were collected from Aznalcázar, near Seville, Spain, between the 8th and 12th of November, 2008. One additional colony used was collected in February 2007 and had undergone laboratory hibernation. Entire colonies were placed in open plastic boxes of 16 × 26 cm, the walls of which were treated with fluon to prevent ant escape, and kept in a constant temperature room (~28°C) under a 12/12 hr day/night light cycle. Colonies were housed in nests made of two plastic Petri dishes of 10 × 5.7 cm each (diameter × height) placed on top of one another. The top Petri dish provided the nesting space while the bottom Petri dish contained water, creating a humid environment in the top box via a thin metallic mesh. After one week, queens were removed from all colonies and workers and brood were divided evenly between two identical nest boxes, creating a total of 60 orphaned groups. One half of each colony was assigned to the control group and the other to the treatment group, and thus control and treatment groups were of identical size. Groups were provided with water and sugar cubes *ad libitum*, and fed freeze-dried crickets and an artificial diet three times a week. Colony size in the initially collected colonies ranged from 550 to 1698 (mean ± SE: 1031 ± 57), with experimental/treatment groups half this size.

In the absence of a queen, *A. senilis *workers rear existing brood into replacement queens, and groups were monitored over the following 18 weeks for developing queen pupae. Queens were weighed and paint marked two days after emergence to permit them time to develop a hardened cuticle and be accepted by workers. In experimental groups, the left mandible of the first emerging queen was ablated at this time using small scissors. Queens were given several minutes rest before being returned to their group. Workers seemed to detect the injury to ablated queens, but behaviour soon returned to normal following reintroduction in both groups. Any sexual (queen) pupae reared after the first two queens had emerged were removed to simplify the selection process.

**Figure 1:**
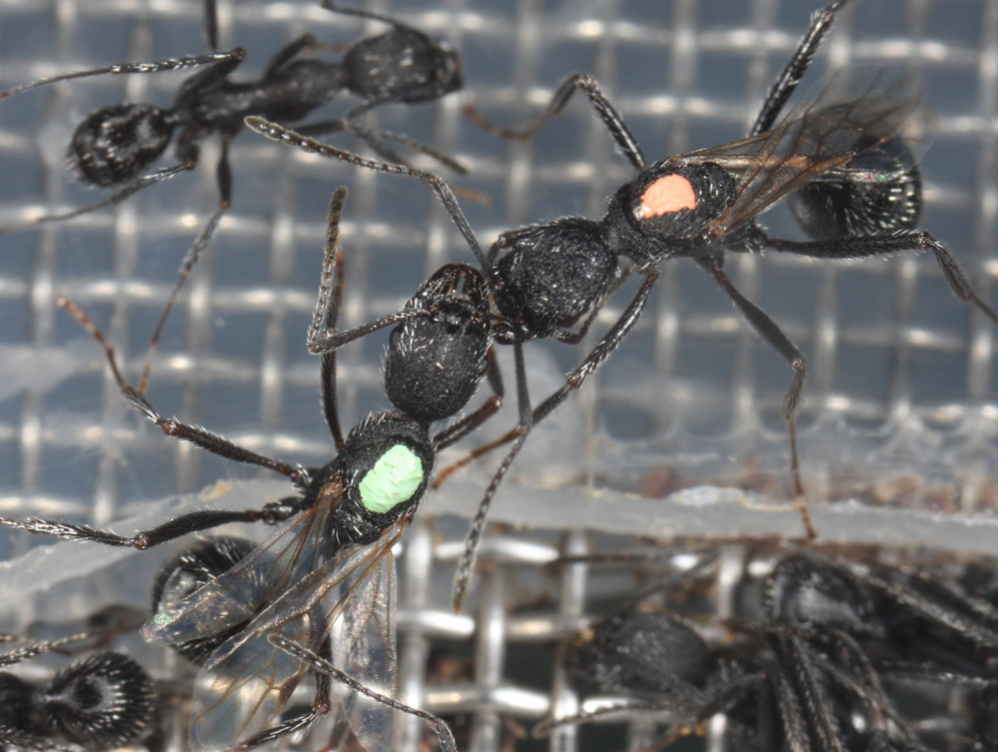
**Queen aggression in *Aphaenogaster senilis***. A first-born queen (orange mark) bites the antenna of a second-born queen (green mark).

Groups were monitored daily for eclosion and survival of queens for the duration of the experiment (18 weeks). In addition, behavioural observations were made for the duration of the association. The scan method [30] was employed as in a previous study of this species [7], with repeated observations made daily. Classification of behaviour followed table 1, and included a behavioural and location component for each observation. Aggression observed included antennation, 'stand-over' and biting (figure 1). The latter was almost always associated with curling of the gaster of the aggressor toward the victim. Although clipped queens had trouble actually biting the victim, they harassed subordinates in the same manner as intact queens. They occasionally managed to grasp narrow appendages such as antennae but in general could not bite effectively. Stand-overs were not necessarily coupled with antennation or biting, and involved the aggressor standing stationary over the victim with legs extended. While antennation was not overtly aggressive, it was usually associated with other dominance behaviours and, as all forms of interaction were usually unilateral (see below), it is included as a measure of dominance here.

**Table 1:** Categories of behaviour and location recorded during scan sampling.

**Behaviour**		**Location**
**Non-aggressive**	**Aggressive**	
Groomed by workers	Antennating	Outside nest
Self-grooming	Biting	In nest not near brood
Stationary	Standing over	In nest near brood
Walking		

### Statistical analyses

Statistics were carried out in the 'R' statistical package, version 2.8.1 for Windows [31]. In the generalised linear model analysis (GLM), the focal variable (which queen was victorious) was binary (queen 1 or queen 2 prevailed) and thus we assumed binomial errors, and began analyses with all explanatory variables fitted (see results). Starting with interaction terms, we then subtracted terms from the model until further removals led to significant (p < 0.05) increases in deviance, as assessed from tabulated values of χ^2^. Significance levels are reported for terms when adding them last to this minimal adequate model. Means are quoted with standard errors throughout unless otherwise noted.

## Results

### Queen replacement

Two-queen associations arose in 54 of 60 orphaned groups (26 treatment and 28 control); four treatment and two control groups (including two treatment-control pairs) did not produce a second queen (hereafter Q2) and were excluded from subsequent analyses. Orphaned groups produced first queens (Q1s) 31.6 ± 2.4 days after being orphaned (range 22-93). In four instances, the first queen died before eclosion of the second queen. This was not a treatment effect as treated queens were no more likely to die than untreated (n = 2 of each). Indeed, treated (ablated) individuals survived the entire observation period (up to 96 days), suggesting no negative effect of the treatment on longevity. In the four cases above monitoring was maintained until further rearing of sexual brood lead to the presence of two queens. Q2s were produced 46.3 ± 3.0 days after orphaning (range 27-111), or 14.7 ± 1.9 days after first queens (range 1-65). Third and subsequent sexual brood were detected (and removed) in 28 groups (number removed 3rd = 15, 4th = 5, 5th = 6, 6th = 2). As pupae were removed before eclosion we cannot assess how this would translate into adults, but these data concur with a previous study in that multiple replacement queens (up to 5) can be produced [7]. Overall, Q1s were significantly heavier than Q2s (paired samples t-test, t = 5.59, p < 0.01, n = 50 as weights were not available for all queens, means = 11.2 ± 1.6 mg for Q1s and 10.1 ± 1.6 mg for Q2s), and Q1 weight was correlated with that of Q2s from the same group (Pearson's r = 0.308, p = 0.03). This difference was significant in both control and treatment groups (t_24 _= 3.59, p < 0.01, and t_22 _= 4.27, p < 0.01 respectively).

### Contest outcomes

Contests were resolved in favour of one or other queen (i.e. a queen died) in 50 groups, 15.9 ± 1.7 days (range 0-51) after the emergence of Q2s. In two other groups both queens were found dead on the same day, and in the last two groups both queens remained alive when the experiment was terminated (after 19 and 71 days of cohabitation). First eclosed queens were significantly more likely to prevail, and did so in 76% of cases overall (n = 50), 78% (n = 23) of treatment and 74% (n = 27) of control groups (χ^2^_1 _= 13.52, p < 0.001 overall; χ^2^_1 _= 6.26, p = 0.012 control; χ^2^_1 _= 7.34, p = 0.007 treatment). GLM analysis with group, queen age, queen weight, date of contest resolution, initial group size, contest duration, treatment and dominance status (see below) as potential explanatory variables indicated that only dominance status had a significant effect (p < 0.001) on the outcome of contests (ie: which queen prevailed).

### Behaviour

**Figure 2:**
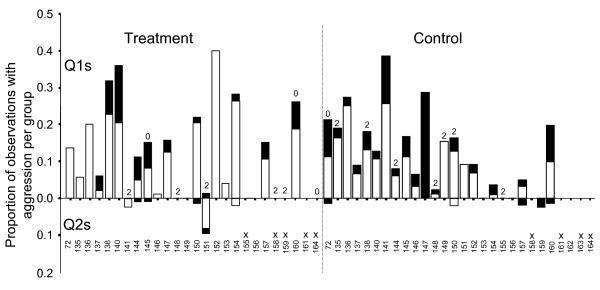
**Mean proportion of observations that were aggression in each group, for Q1s (above) and Q2s (below)**. Bars are divided into black for antennations and white for bites and stand-overs. Q1s prevailed in all groups except where indicated by numerals above bars (2 = Q2 won, 0 = both queens died or there was no result). Colony numbers are given at the bottom, with x indicating there were no behavioural data available for that group.

We performed a total of 2297 scans over 88 days from 45 groups (mean per group 51.3 ± 5.6; range 2 to 219; from 24 control and 21 treatment groups). No behavioural observations were possible in 9 groups as queens were killed before observations. We observed a total of 296 aggressions (antennation, stand-over or bite; including extended bouts), as summarised in figure 2. Aggression was scored as the proportion of scans in which a given queen was exhibiting aggressive behaviour (see table 2). Q1s were aggressive in a higher proportion of scans than Q2s overall (paired samples t-test, t_44 _= 6.97, p < 0.001) and in treatment and control groups (t_20 _= 4.49, p < 0.001, and t_23 _= 5.40, p < 0.001 respectively). Aggression did not differ between treatments for Q1s or Q2s (t-tests: t_43 _= 0.60, p = 0.55, and t_43 _= 0.95, p = 0.35 respectively). No aggression between queens was observed in 7 groups prior to queen selection. In the remaining 38 groups (where dominance could be ascertained), Q1s were behaviourally dominant in all but 3 cases (92%). Q1s were equally likely to be dominant in control and treatment groups (Fisher's Exact test: p = 0.595, n = 38). Q2s emerged victorious in 12 instances overall and behavioural data were available for 10 of these. Q1 was behaviourally dominant in 6 cases, Q2 was dominant in 2 cases, and there was no aggression in the final 2 cases (figure 2). This indicates that dominance did not guarantee success, though dominant queens prevailed in 73% (n = 35) of cases where dominance could be ascertained and one queen prevailed over the other.

**Table 2:** Mean (± SD) aggression for each queen for different treatment groups and outcomes.

		**Q1 wins**	**Q2 wins**	**No winner**	**Overall**
Treatment	Q1	0.157 ± 0.131 (16)	0.005 ± 0.008 (3)	0.206 ± 0.078 (2)	0.140 ± 0.129 (21)
	Q2	0.003 ± 0.006 (16)	0.039 ± 0.050 (3)	0.002 ± 0.003 (2)	0.008 ± 0.021 (21)
Control	Q1	0.116 ± 0.117 (16)	0.113 ± 0.078 (7)	0.211 (1)	0.119 ± 0.104 (24)
	Q2	0.003 ± 0.007 (16)	0.003 ± 0.007 (7)	0.014 (1)	0.004 ± 0.007 (24)
Overall	Q1	0.136 ± 0.124 (32)	0.080 ± 0.083 (10)	0.208 ± 0.055 (3)	0.129 ± 0.115 (45)
	Q2	0.003 ± 0.007 (32)	0.013 ± 0.030 (10)	0.006 ± 0.007 (3)	0.006 ± 0.015 (45)

Aggression (proportion of scans where queens exhibited aggressive behaviour) = antennation, stand-over and biting; sample sizes (number of groups) are given in parenthesis. See text for statistics and further information.

**Figure 3:**
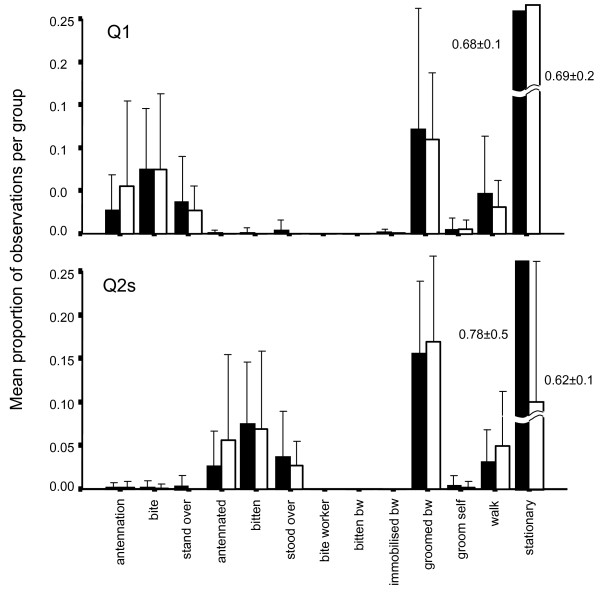
**Mean proportion (± SD) of observations in each behavioural category per group, for Q1s (above) and Q2s (below)**. White bars represent control groups whereas black bars represent treatment groups.

Behavioural profiles for Q1s and Q2s are summarised in figure 3, and indicate that treatment had no influence on the general behaviour of queens. Indeed, behaviour of both Q1s and Q2s did not differ between controls and treatments. Q1s differed from Q2s in interactions involving dominance and in the lower proportion of time spent being groomed by workers, but were otherwise similar. Queen location data are summarised in table 3, and indicate no variation between treatment and control groups. There was a strong difference between Q1s and Q2s however, with Q2s spending a higher proportion of time inside the nest. This is likely to be the result of the age difference between Q1and Q2, with Q1 seeking mating outside of the nest while Q2 (which is younger, subordinate, and probably sexually immature) remains in the nest. This difference may also stem from an observation bias, because Q1s were not monitored until Q2s eclosed and thus early post-eclosion behaviour was missed.

**Table 3:** Mean (± SD) percentage of time queens spent in different locations during behavioural observations.

		**In nest near brood**	**In nest not near brood**	**Outside nest**
Treatment	Q1	32 ± 21	39 ± 11	29 ± 17
	Q2	61 ± 22	30 ± 17	8 ± 11
Control	Q1	36 ± 23	41 ± 18	23 ± 18
	Q2	61 ± 30	31 ± 20	9 ± 16

(n = 21 Treatment and 24 Control groups).

Subordinates were completely stationary and often assumed a crouched posture or lay flat during biting or stand-overs, and occasionally during antennation. At no time were subordinates observed attempting to retaliate, defend themselves or escape when aggressed by a dominant. Bouts of biting could last many minutes (fights > 35 mins observed), during which time the dominant queen would attack the subordinate repeatedly, often interspersed with short periods of walking away from the subordinate. In contrast, worker aggression occurred only during queen execution. Several executions were observed in which multiple workers (up to around twenty) would immobilise and bite the queen, continuing until the victim was dismembered and eventually devoured. Victims did attempt to flee from workers when under attack, but were never successful. Queens were generally not observed aiding in queen executions and queen aggression did not appear to pre-empt queen execution. The one exception to this was an instance where Q1 was observed immobilising a Q2 with the aid of three workers for ~1 hr before leaving workers, who then executed Q2 (which required a further 3 hrs). Queen aggression never otherwise included immobilisation, except in one further instance in which Q1 was immobilised for several minutes during the execution of Q2.

## Discussion

Queen selection is a critical event in the life of a colony. It represents not only a potential conflict of interest between queens, workers, and the colony as a whole, but also a possibility for colony failure. Workers are known to be responsible for selection of queens in several species of ants, and cull queens based on a range of criteria [1]. It is in the interests of queens, however, to improve their chances of survival directly by fighting among themselves or indirectly through improvement of characters on which workers may select queens, such as reproductive readiness. A previous study [7] of queen replacement in *Aphaenogaster senilis *indicated that first-born queens were behaviourally dominant over subsequently produced queens and usually survived the queen selection process. Chéron et al [7] proposed that additional queens were produced as insurance against death or shortcomings of the first queen, and that queen selection was based on order of eclosion, presumably to minimise the delay to reproductive activity. They further suggested that production of insurance queens was delayed to permit first queens every advantage in selection, minimising chances of colony failure from both queens being injured during conflict.

Our results are similar with respect to timing of queen replacement and number of queens produced, and concur with Chéron et al [7] in that first-born queens were generally dominant and survived in the majority of cases. The importance of order of eclosion is reinforced by the lack of any effect of mandibular ablation on contest outcome or queen behaviour. However, in contrast to their results, our data show that while dominant individuals were more likely to succeed overall, dominance does not guarantee success, and behaviourally dominant queens occasionally lose contests. In addition, we also demonstrate that Q2s can be behaviourally dominant over Q1s. Finally, the finding that mandibular ablation has no effect is in marked contrast to the impact of this treatment in honeybees, where ablated queens actively avoid conflict [29].

While dominance is important for survival in queens of *A. senilis*, the proximate mechanism triggering queen execution remains unclear. Indeed, disentangling worker and queen roles in queen elimination can be problematic: workers attack already injured queens in *Solenopsis invicta *[15,16,32], and queen elimination by workers was not significantly distinct from queen-queen aggression in *Messor *(*Veromessor*)*pergandei *[33]. The long duration of cohabitation after fighting bouts in *A. senilis *argues against queens using chemical means to 'mark' subordinates for execution or immobilisation (eg: [34,35]) and no behaviour that could be construed as marking was observed. Aggression can have direct affects (eg: injury) or indirect affects (eg: ovarian suppression). Mandibular ablation in *A. senilis *clearly affected the capacity to fight as queens predominantly use biting as a means of aggression, and ablated queens had difficulties grasping opponents. Several factors however, point to a lack of any direct affect of fighting in contest resolution. Firstly, the duration of queen cohabitation was prolonged (mean 16 days) and did not differ between control and treatment groups. We witnessed multiple bouts of aggression in some groups, some of which were extended (>35 minutes), yet at no time did this clearly result in an injury to either queen or lead to queen culling. In contrast, queen contests in honeybees are resolved fatally and relatively quickly (4s-15 mins [36]). Secondly, whereas mandibular ablation had a profound effect on the behaviour of honeybee queens, which avoided contests when ablated [29], the behavioural profile of *A. senilis *queens did not differ between treatments. This suggests mutilated Q1s did not detect any reduced chance of victory despite having ample opportunity to assess their condition during bouts. In addition, Q2s did not behave differently when aggressed by a mutilated versus an un-mutilated Q1, suggesting that they did not assess the mutilated Q1s as weakened. Finally, aggression was always unilateral: subordinates never fought back or attempted to escape (whereas they did flee from mass worker aggression) suggesting they did not consider themselves under direct threat from queen aggression. These data suggest that aggression in *A. senilis *is ritualised and serves primarily as a dominance display rather than a direct means of resolving contests. This may explain the different response to ablation in honeybees, where fighting between queens is the primary means of contest resolution and bouts are usually fatal [3].

Dominance is important in reproductive success in many species, and can be correlated with productivity or fitness [2]. In *Leptothorax *sp. A dominance was correlated with ovarian status and attractiveness to workers, and lead to preferential feeding and eventual expulsion or killing of subordinates [37,38]. We did not see preferential feeding, but differential treatment of Q1 and Q2 may be indicated by lower observed allogrooming of Q2s. Aggression probably acts to suppress the development of characters upon which worker selection acts, such as reproductive ability or pheromone production [39,40]. The observation of one apparently healthy queen being attacked and killed by workers supports the idea of worker mediated queen culling. Yet if workers are controlling selection and presumably would favour rapid resolution of contests [2,8], this raises the question of why some contests are so prolonged. One explanation is that asymmetries between queens take time to develop and workers are waiting for an asymmetry threshold to be reached. Differential ovarian development and chemical signalling of this condition is one possibility (eg: [41]). Development of ovaries and signalling of this condition takes around 6-8 weeks in the ponerine ant *Dinoponera quadriceps *[42], which is considerably longer than our mean coexistence time of 16 days. Worker awareness of ovarian development has been demonstrated in *Aphaenogaster cockerelli *and workers kill "cheating" (laying) workers [43,44]. Another possibility is that workers await one queen to be mated before culling the other queens. As mating did not occur in the laboratory, this could result in longer co-inhabitation of queens than under natural conditions. Q1s have a distinct head start in development of any such characters, and Q2s can only prevail presumably if Q1 is of relatively poor fitness in general. These data support the "supernumerary queens as life insurance" hypothesis of Chéron et al [7] and indicate that, rather than fighting ability, the process of queen selection is based on advantages relating to acquired asymmetries, which are in turn at least partially associated with order of eclosion. Aggression based dominance may aid first-born queens in the contest to reach the acquired asymmetry threshold that triggers worker intervention, but aggression is probably not directly involved in contest resolution.

Aggression is, however, important in the establishment of dominance, and a problem that remains is explaining the lack of aggression from subordinate queens. Although some subordinate queens were able to prevail, dominant queens succeeded in 73% of cases for which dominance could be ascertained. Queens produced during emergency queen production in *A. senilis *have few options other than inheriting the colony, as dispersal is only possible through colony fission which can only occur in large colonies and under certain conditions [5]. Thus, not fighting may effectively be a form of suicide for Q2s. Reduced aggression in animal contests is often associated with the use of conventions to decide contests, which act to remove or minimise the risks of overt fighting [45]. Prior residency is one convention that is common in a range of animal taxa [46], though the mechanisms underlying the effect have been the subject of much debate [47]. The prior residency effect operates when challengers for a resource back down in favour of residents, as in the 'bourgeois' strategy of game theory [9,48]. For example, in the parasitoid wasp *Trissolcus basalis*, the first arriving female at a patch of host eggs is more likely to win contests than intruders, who back down before escalated conflict [49]. Similarly, first-born queens in *A. senilis *are effectively prior residents, and in most cases this translated to behavioural dominance and eventual victory. Queens are thus basically following a 'bourgeois' convention. However, game theory models also predict that when the value of the contested resources represents the majority of an individual's possible lifetime fitness, contestants should fight regardless of ability, kinship or asymmetries [50-52]. Hence, while asymmetries may discourage lesser individuals from overt fighting, fighting should occur regardless when reproductive alternatives are scarce or non-existant. For example, queens of *Apis mellifera *with one mandible ablated avoid contests, but will fight if challenged [29]. Our data suggest that subordinate queens are almost never aggressive, despite the high value of the contested resource and the very low success of subordinates. One aspect of the biology of *A. senilis *that may be important is the high relatedness between queens, which are full sisters (r = 0.75; [7]). In this case a loser can still gain considerable *indirect *fitness even if it is killed, and this indirect fitness may be greater than the benefits of fighting for inheritance if fighting entails risk to both queens. By obeying a bourgeois strategy and ceding to first-born queens, second-born queens can minimise the chance of both queens being injured while also facilitating rapid commencement of reproductive activity in the colony.

## Conclusion

Queen selection is an important process in the lifecycle of a colony as it influences the fitness of all colony members. First-born *Aphaenogaster senilis *queens were behaviourally dominant over second-born queens, and inherited the colony in most cases. Ablating one mandible of the first-born queen did not affect contest outcomes, suggesting aggression is probably a form of ritualised dominance. Queens are likely selected by workers based on reproductive quality, with first-born queens gaining a significant time-related advantage over subsequently-born queens. Queens may follow a bourgeois strategy in contests, with second-born queens effectively committing suicide by ceding to first-born queens. This minimises the chance of colony failure and reproductive delay through injury or death of both queens, and is possible because queens are full sisters and thus highly related.

## Competing interests

The authors declare that they have no competing interests.

## Authors' contributions

Both authors conceived of the study, designed and executed the experiment, and contributed to the final manuscript. ALC performed analyses and drafted the manuscript.
